# Left ventricular pseudo-false aneurysm perforating the right ventricle: two case reports

**DOI:** 10.1186/s44215-023-00108-4

**Published:** 2023-08-29

**Authors:** Hiroki Moriuchi, Masaaki Koide, Yoshifumi Kunii, Minori Tateishi, Takuya Maeda, Kumiko Sone

**Affiliations:** Department of Cardiovascular Surgery, Seirei Hamamatsu Hospital, Shizuoka, Japan

**Keywords:** Left ventricular pseudo-false aneurysm, Perforate into the right ventricle

## Abstract

**Background:**

Left ventricular (LV) pseudo-false aneurysm is a rare and fatal complication secondary to myocardial infarction. Sometimes, it may perforate the right ventricle (RV) and lead to acute heart failure. We experienced 2 cases of surgical repair of an LV pseudo-false aneurysm perforating the RV.

**Case presentation:**

Case 1: A 76-year-old man was referred to our hospital due to dyspnea. Echocardiography revealed an LV pseudo-false aneurysm (25 mm × 20 mm) that had perforated the RV. Via LV incision, the two small orifices communicating to the RV were detected and closed by direct suture. A double patch of bovine pericardium and a dacron sheet was sutured around the aneurysm with everting mattress. Case 2: A 51-year-old man, who had undergone percutaneous coronary intervention 1 month before, was referred to our hospital due to an LV aneurysm perforating the RV. Via LV incision, a double-layered patch was sutured around the aneurysm with everting mattress. The communicating hole to RV was closed by bovine pericardium patch with a running suture via an RV incision. Postoperative course was uneventful in both cases.

**Conclusions:**

An LV pseudo-false aneurysm perforating the RV should be considered for urgent repair before serious complications arise and the patient’s general condition deteriorates.

## Background

Ventricular septal perforation (VSP) is a serious and potentially fatal complication after myocardial infarction (MI). VSP with pseudo-false aneurysm in the acute phase after MI is a rare but potentially fatal condition. We report two cases of left ventricular (LV) pseudo-false aneurysm perforating into the right ventricle (RV).

## Case presentation

### Case 1

**Fig. 1:**
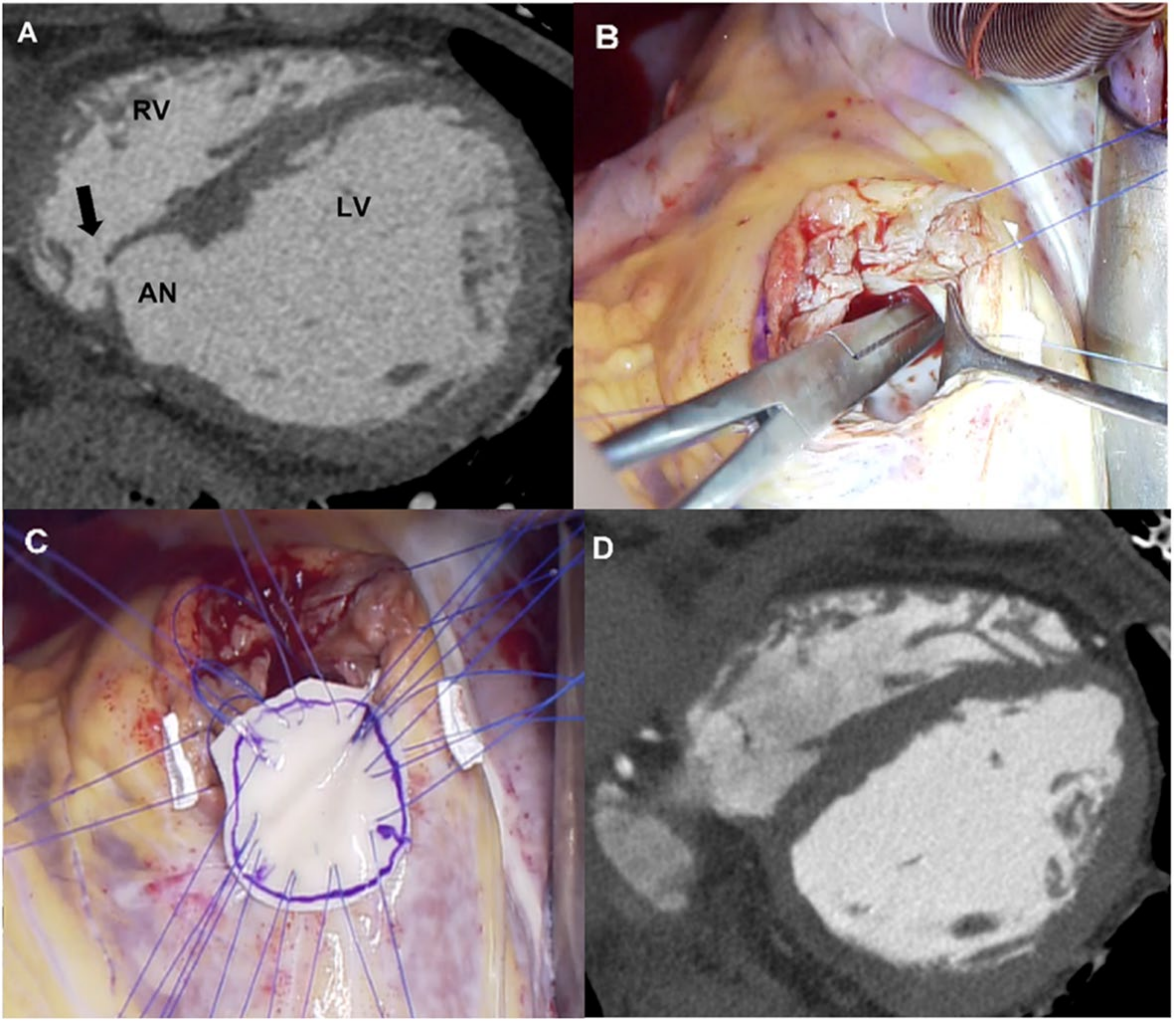
**A** Computed tomography (CT) showing a 25 × 15 mm left ventricular pseudo-false aneurysm AN). The black arrow indicates the orifice communicating with right ventricle. **B** and **C** The forceps are shown in the right ventricle through the small orifice from aneurysm. Aneurysm was closed with a bovine pericardium patch and polyester cloth with interrupted mattress suture. **D** Postoperative CT shows the absence of both the aneurysm and residual shunt

A 76-year-old man was admitted to a local hospital with dyspnea on exertion and treated for 1 week. Because echocardiography revealed an LV aneurysm communicating to the RV, he was referred to our hospital. Echocardiogram showed that the LV end-diastolic diameter (LVDd) was 56 mm, and ejection fraction (EF) was 55%. Cardiac catheterization showed mean pulmonary artery pressure was 26 mmHg, and pulmonary/systemic flow ratio was 2.5. Coronary angiography showed 90% stenosis in the proximal right coronary artery (RCA) and no stenosis in the left coronary artery. Contrast-enhanced computed tomography (CT) showed a 25 mm × 20 mm aneurysm on the LV posterior wall perforating into the RV (Fig. 1A). After controlling heart failure with medication, we performed patch repair of the pseudo-false aneurysm and closure of the perforation. Under general anesthesia, median sternotomy was performed. Cardiopulmonary bypass (CPB) was established, and an LV vent was placed via the right upper pulmonary vein. Following cardiac arrest by antegrade and retrograde infusion of cardioplegia, we lifted the heart to expose the postero-basal aspect of the ventricle. A longitudinal incision of LV along the posterior descending artery (PDA) was made. There was no adhesion between LV wall around the aneurysm and the surrounding tissue. Two small orifices communicating to the RV were detected, and we closed them by direct suture. A double patch of bovine pericardium and a dacron sheet was sutured around the aneurysm with everting mattress (Fig. 1B, C). The fibrosis around the aneurysm was well advanced and tough; suturing was easy. The incision was closed with running suture using Teflon felt strips. The operative schema was seen in Fig. 3A. Postoperative CT revealed exclusion of the aneurysm and no residual shunt (Fig. 1D). The patient was discharged 14 days after surgery.

### Case 2

**Fig. 2:**
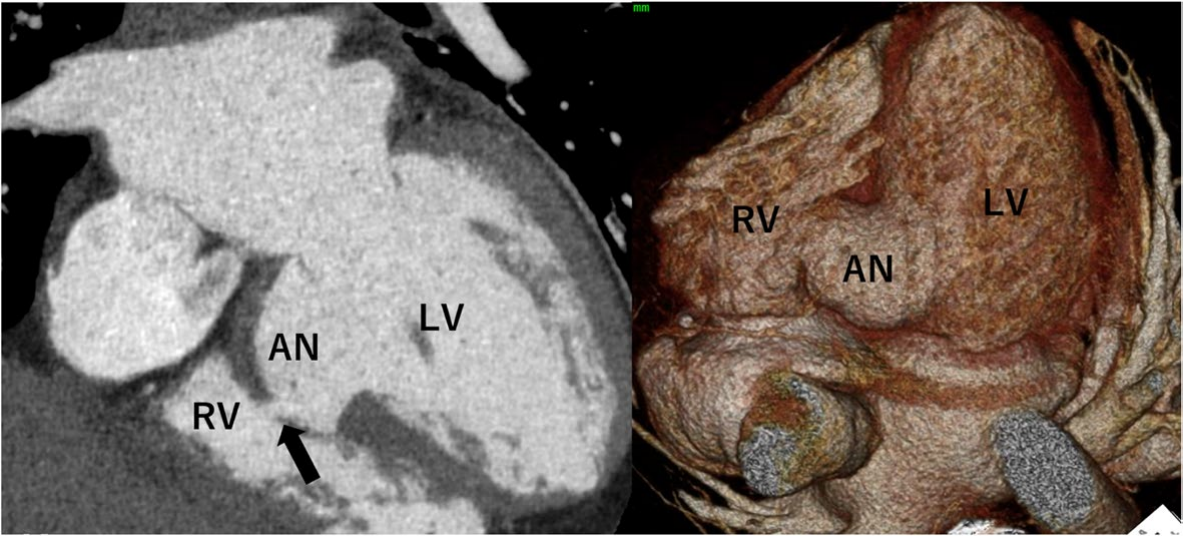
CT shows a 1 × 30 mm aneurysm (AN) on the LV posterior wall. The black arrow indicates perforation into the RV

**Fig. 3:**
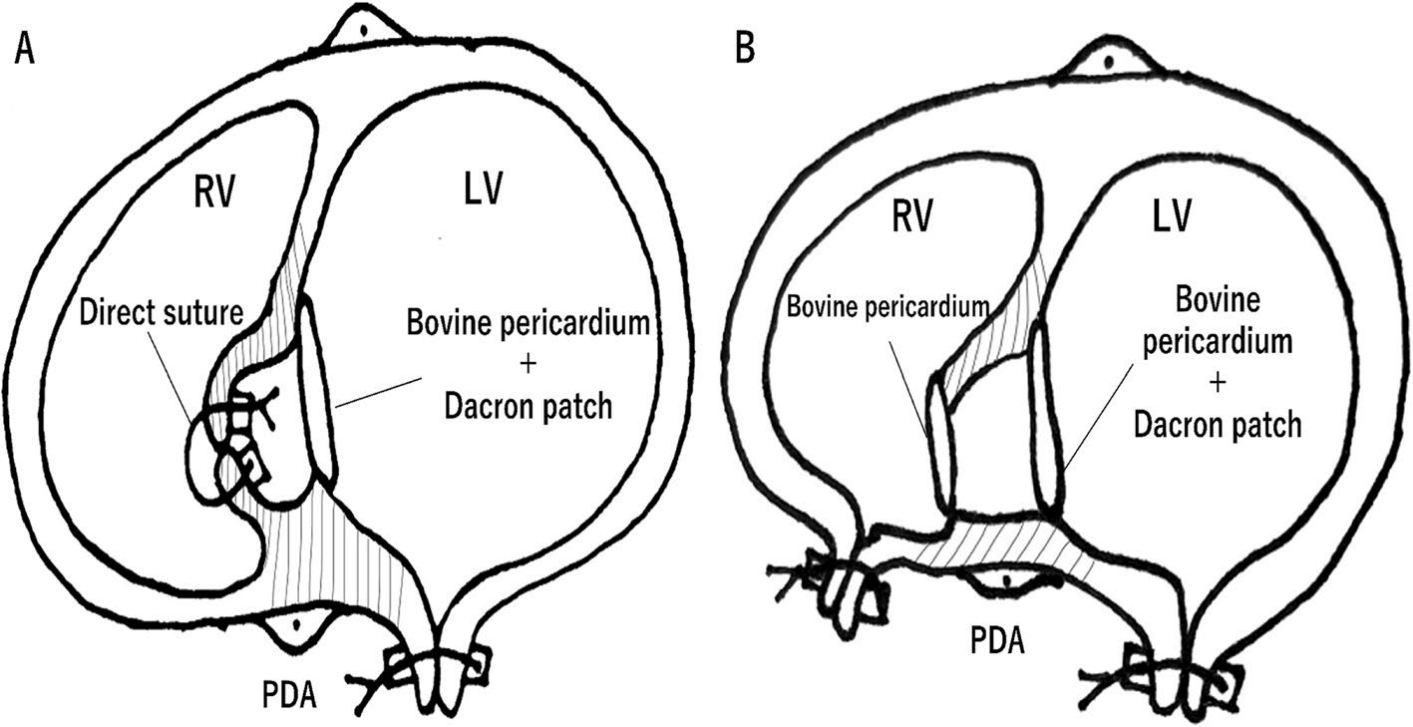
**A** Operative schema of case 1. The orifice on the RV side was small and could be closed by direct suture. **B** Operative schema of case 2. The orifice on the RV side was large, and RV incision was necessary to perform patch closure with a good field of view

A 51-year-old man, who underwent percutaneous coronary intervention to mid-RCA for acute MI 1 month before, was referred to our hospital due to an LV aneurysm perforating into the RV. He had dyspnea, and chest X-P showed pulmonary congestion. Echocardiography revealed that the LVDd was 55 mm and EF was 53% and blood flow passing from the aneurysm to the RV. Right heart catheterization showed mean pulmonary artery pressure was 43 mmHg, and pulmonary/systemic flow ratio was 3.0. CT showed a 15 × 30 mm aneurysm on the LV posterior wall perforating into the RV (Fig. 2). Because heart failure was progressive even under medication, we decided to perform an urgent operation. CPB was established with an ascending aorta and bicaval cannulation; cardiac arrest was achieved by antegrade and retrograde infusion of cardioplegia. A longitudinal incision was made on LV along the PDA. No adhesion between the LV aneurysm and the pericardium was seen. The aneurysm was 20 × 30 mm, and the diameter of the communicating hole to the RV was 15 mm. A double patch of bovine pericardium and a dacron sheet was sutured around the aneurysm with everting mattress. The incision was closed with running suture using Teflon felt strips. The right side of the PDA on RV was incised, and the communicating hole to the RV was closed by bovine pericardium patch with a running suture. The RV incision was closed with the same maneuver as for the LV. The operative schema was seen in Fig. 3B. Postoperative cardiac echo revealed no residual shunt, and he was discharged 12 days after surgery.

## Discussion and conclusions

LV pseudo-false aneurysm, which was first reported by Stewart et al. [1], is a rare complication secondary to MI. It occurs when an intramyocardial dissecting hematoma does not completely reach the epicardium and is contained within the area of the infarcted myocardium. LV pseudo-false aneurysm can occur from a few weeks to a few years after initial MI. Inoue et al. [2] and Tasaki et al. [3] reported the perforation of an LV pseudo-false aneurysm into the RV, and they described it as an extremely rare condition. Osawa et al. [4] reported that pseudo-false aneurysm tends to be formed in the posterior and lateral wall of the LV, where the ventricular muscle band is composed of only one layer. The muscle layer crosses at the anterior of the LV forming a double layer. LV pseudo-false aneurysm perforating into the RV may worsen hemodynamics when shunt flow is large and have a higher possibility of rupture compared with a LV true aneurysm [4]. Therefore, LV pseudo-false aneurysm should be repaired immediately.

In both of our cases, pathological examination was not performed. Our diagnosis of LV pseudo-false aneurysm was based on operative findings of no adhesion between the epicardium and the pericardium. Preoperative CT revealed precisely the location of aneurysm and coronary artery; the incision site could be decided before operation.

When the orifice is small, it can be closed directly, but when the orifice is large, it should be closed with a patch so as not to distort the LV and the coronary artery. In case 1, the orifice communicating with the RV was directly closed, and the orifice communicating with the LV was closed with a patch. In case 2, both orifices were large, so patch closure was performed for both. RV incision was necessary to close the orifice on the RV side with a good field of view. Patch closure on the LV side only is not a complete procedure, because without patch closure on the RV side, blood flow to the aneurysm will remain and increase the probability of shunt recurrence in the remote phase. The objectives of this surgery are to exclude the aneurysm and eliminate the shunt flow, so we performed patch closure on the LV and RV side. In both cases, no residual shunt or distortion of the LV was observed.

Inoue et al. [2] and Tasaki et al. [3] described the perforation of LV pseudo-false aneurysm into the RV after MI in the chronic phase. However, in the present cases, it occurred in the acute or subacute phase after MI, which is extremely rare. In addition to the follow-up of coronary lesions, attention must also be paid to the occurrence of mechanical complications such as pseudo-false aneurysm. Surgical repair is necessary in cases of hemodynamic instability due to shunt.

## Data Availability

The data underlying this article will be shared on reasonable request to the corresponding author.

## Abbreviations

LV: Left ventricle
RV: Right ventricle
VSP: Ventricular septal perforation
MI: Myocardial infarction
LVDd: Left ventricular end-diastolic diameter
EF: Ejection fraction
PDA: Posterior descending artery
AN: Aneurysm
